# Inflorescence photosynthetic contribution to fitness releases *Arabidopsis thaliana* plants from trade-off constraints on early flowering

**DOI:** 10.1371/journal.pone.0185835

**Published:** 2017-10-03

**Authors:** Sebastian Gnan, Tom Marsh, Paula X. Kover

**Affiliations:** Milner Centre for Evolution, Department of Biology and Biochemistry, University of Bath, Claverton Down, United Kingdom; Michigan State University, UNITED STATES

## Abstract

Leaves are thought to be the primary carbon source for reproduction in plants, so a positive relationship between vegetative size and reproductive output is expected, establishing a trade-off between time to reproduction and reproductive output. A common response to higher temperatures due to climate changes is the induction of earlier transition into reproduction. Thus, in annual plants, earlier transition into flowering can potentially constrain plant size and reduce seed production. However, trade-offs between early reproduction and fitness are not always observed, suggesting mechanisms to escape the constraints of early flowering do exist. Here, we test whether inflorescence photosynthesis contribution to the reproductive output of *Arabidopsis thaliana* can offset the cost of early reproduction. We followed the development, growth rate and fitness of 15 accessions, and removed all rosette leaves at flowering (prior to the completion of inflorescence development or any fruit production) in half of the plants to determine the ability of inflorescences to maintain fitness in the absence of leaves. Although leaf removal significantly reduced fruit number, seed weight and plant height, even the most severely impacted accessions maintained 35% of their fitness with the inflorescence as the sole photosynthetic organ; and some accessions experienced no reduction in fitness. Differences between accessions in their ability to maintain fitness after leaf removal is best explained by earlier flowering time and the ability to maintain as many or more branches after leaf removal as in the control treatment. Although earlier flowering does constrain plant vegetative size, we found that inflorescence photosynthesis can significantly contribute to seed production, explaining why early flowering plants can maintain high fitness despite a reduction in vegetative size. Thus, plants can be released from the usually assumed trade-offs associated with earlier reproduction, and selection on inflorescence traits can mediate the impact of climate change on phenology.

## Introduction

An organism’s life-history is shaped by its allocation to growth, maintenance (e.g. tissue repair and resistance to pathogens) and reproductive functions. Because resources are usually limited, life-history theory expects trade-offs between its allocation to different functions [1, 2]. Assuming all else remains the same, an earlier transition into reproduction is thought to restrict plant vegetative size and consequently the resources available for offspring production, reducing the quality and/or number of progeny [3,4,5,6]. Trade-offs between flowering time and vegetative size are particularly important for annual plants, because they have a single chance at maximizing fitness. There is concern that changes in the global climate may affect plant populations’ persistence and crop yield by causing many annual plant species to flower earlier [7,8,9,10]. Thus, it is important to determine whether there are mechanisms that can release annual plants from the expected trade-off between earlier flowering and fitness (yield).

Annual plants are a particularly good subject to investigate trade-offs between growth, time to reproduction, and fitness because they have distinctive vegetative and reproductive phases. A positive relationship between vegetative size and reproductive output is expected because the vegetative leaves are assumed to be the primary photosynthetic tissue responsible for carbon acquisition. Correlations between flowering time and vegetative size have been observed in a number of plants [11,12], as well as between vegetative size and reproductive output [12, 13]. However, many exceptions to the correlation between flowering time and reproductive output have also been observed [14, 15,16,17,18]. For example, Kover et al [19] compared ancestral and derived populations of *Arabidopsis thaliana* selected to flower earlier, and found that derived early flowering populations showed no reduction in fruit production.

A couple of mechanisms have been proposed to explain how reproductive output may be decoupled from time to reproduction. One hypothesis is that variation in the growth rate can remove the relationship between age of reproduction and plant size [2,18,20]. Thus, earlier flowering plants may also be larger if they have a larger growth rate (i.e. leaf production over time); and therefore, have more photosynthetic resources than plants that grow slowly and reproduce later. Genetic variation in growth rate has been previously observed within plant species [14, 18, 21, 22] but its correlation with flowering time and fitness in the context of understanding trade-offs or its absence has not been considered.

Alternatively, it is possible that the reproductive structure itself (inflorescence and flowers) contributes significantly to carbon acquisition. Green reproductive structures have previously been shown to be photosynthetically active and contribute to overall carbon gain [23,24,25,26,27,28]. If this is the case, an earlier transition to flowering can increase the resources available for future reproduction by increasing photosynthetic surfaces to support reproduction; releasing plants from the evolutionary constraints posed on flowering time evolution by the trade-off between age and size at reproduction. However, how much of a plant’s reproductive output can be maintained by the inflorescence photosynthetic ability is not well known.

Significant variation in flowering time, development rate and reproductive output has been previously observed in the annual plant *A*. *thaliana* [22,29]. The development of *A*. *thaliana* is divided into a vegetative phase where a laminar rosette grows in terms of leaf number and leaf area, and a reproductive phase characterised by an inflorescence developing vertically. It has been shown that the inflorescence of *A*. *thaliana* contributes significantly to carbon gain [27,28]. Earley *et al*. [27] also showed that the contribution of the inflorescence to carbon gain varies among natural accessions, and suggested that this variation may be an adaptive response to the accession’s climate of origin. They propose that switching to higher photosynthetic activity in the inflorescence may be favoured in warmer climates, where the increased ambient temperature at the soil surface makes photosynthesis in the rosette leaves less efficient.

Here, we investigate whether the inflorescence photosynthetic activity in *A*. *thaliana* can maintain seed production (fitness). We use a manipulative approach of removing all rosette leaves when plants transition into reproduction. This approach leaves the inflorescence as the sole remaining photosynthetic organ in the treated plants prior to the development of branches or fruits. Control plants are grown simultaneously, where leaves are kept throughout the plant’s life-cycle. Comparison of reproductive output in treatment and control plants of 15 natural accessions of *A*. *thaliana* from a wide geographical range and different flowering times allows the determination of whether there is natural variation in the ability of inflorescences to maintain fitness.

We also consider three specific hypotheses: 1) We hypothesize that if Early *et al*. [27] suggestion is correct, accessions from warmer climates should have inflorescences selected to contribute more to carbon gain, and therefore should be able to maintain a higher proportion of the fitness. 2) Alternatively, it is also possible that early flowering accessions would have been selected to have inflorescences with higher potential to photosynthesize. This could be because either they have to compensate for the smaller rosette size (if there is a positive relationship between flowering time and rosette size), or because ecological conditions that favour earlier flowering such as heat and drought will also favour inflorescence photosynthesis [27,30]. 3) Finally, it is also possible that variation in fitness reduction upon removal is a function of inflorescence size, since photosynthesis capability will increase with more branches and taller inflorescences. To test these hypotheses we will determine whether there is natural variation in the ability of inflorescences to maintain fitness, and whether this variation is correlated with average temperatures in the geographical locations of origin of the accessions used, flowering time, rosette or inflorescence size.

## Materials and methods

### Plant material and growing conditions

Seeds from the 15 natural accessions of *A*. *thaliana* listed on Table 1 were originally obtained from the *Arabidopsis* stock centre (www.arabidopsis.org), and selfed twice in our lab prior to use in these experiments. These accessions were chosen because of their wide geographic distribution, and large genetic variation [31]. Also, these accessions are among the parents of the MAGIC mapping lines [32].

**Table 1:** Rosette growth rate measured as number of rosette leaves per day, at 23 days after planting (when plants were between 18 ad 20 days), and at flowering time (when plants have completed their vegetative growth), averaged across 10 plants per accession.

Accession	Stock #	Origin	Latitude	Spring T	Total Leaves		Growth rate @18–20 days		Growth rate @flowering		Days to Flowering	
					Mean	SE	Mean	SE	Mean	SE	Mean	SE
Bur-0	CS6643	Ireland	53N	4.5	25.9	1.64	0.64	0.01	0.86	0.05	35.6	0.34
Col-0	CS6673	USA	38N	15.5	14.8	0.65	0.53	0.02	0.58	0.04	32.8	0.36
Ct-1	CS6674	Italy	37N	13.5	14.2	0.84	0.54	0.03	0.58	0.04	32.6	0.50
Hi-0	CS6736	Netherlands	52N	5.5	12.8	0.49	0.58	0.02	0.57	0.02	28.6	0.45
Kn-0	CS6762	Lithuania	54N	3.5	16.4	0.83	0.60	0.02	0.65	0.03	32.5	0.67
Ler-0	CS20	Germany	52N	3.5	10.6	0.37	0.50	0.02	0.48	0.02	28.0	0.40
Mt-0	CS1380	Libya	32N	15.5	18.4	1.01	0.69	0.02	0.75	0.04	32.8	0.49
No-0	CS6805	Germany	51N	5.5	12.1	0.48	0.52	0.03	0.50	0.02	31.2	0.53
Oy-0	CS6824	Norway	60N	3.5	15.9	0.28	0.69	0.02	0.66	0.02	32.5	0.27
Rsch-4	CS6850	Russia	56N	1	17.2	0.07	0.65	0.04	0.66	0.42	33.1	0.81
Sf-2	CS6857	Spain	41N	11.5	32.0	4.19	0.58	0.02	1.0	0.11	37.8	1.86
Tsu-0	CS6874	Japan	34N	9.5	26.2	1.01	0.71	0.2	0.93	0.04	35.8	0.51
Wil-2	CS6889	Russia	55N	1	13.5	0.27	0.63	0.01	0.61	0.01	30.0	0.26
Ws-0	CS6891	Russia	52N	3.5	24.0	1.97	0.67	0.01	0.86	0.04	34.3	1.35
Wu-0	CS6897	Germany	49N	5.5	16.9	0.86	0.59	0.03	0.70	0.03	33.2	0.79

Ten replicates of each accession were grown individually in 5.5 cm diameter pots filled with Levingtons F2+S compost (Scotts, Marysville,OH). Pots were randomly allocated to trays and trays were rotated around the greenhouse at regular intervals to homogenize possible microenviromental effects. The experiment was carried out in the University of Bath greenhouse, set at 21°C day/18°C night and 16 hours of light/day. Seeds were suspended in a 0.015% agar solution at 4°C for 3 days to promote simultaneous germination, before being pipetted onto the pot’s surface for germination.

Half of the replicates of each accession were randomly assigned to either the “Removal” or “Control” group. The control group was left to grow normally, while the removal group had all rosette leaves removed using scissors on the day the first flower opened (prior to any seed being developed, and when the inflorescence was still small and unbranched). All removed leaves were attached to a piece of paper and scanned for later estimation of total rosette leaf area using ImageJ (http://rsbweb.nih.gov/ij/).

Plants were inspected every couple of days and the timing of germination, flowering (day first flower opened) and senescence were noted. Senescence was defined as the day when no more open flowers were visible. Prior to removal of leaves, rosette leaves were counted on plants assigned to both treatments every couple of days until flowering (starting 10 days after planting). After the plants had senesced the inflorescence height was measured and the number of branches (derived from the inflorescence or rosette) and fruits per plant were recorded. To assess seed weight and seed number per fruit, 3 fruits between the 6^th^ and 10^th^ fruit on the main stem were collected. A Nikon SMZ 1500 dissecting microscope with a Nikon Digital Sight DS-U1 camera was used to capture images of seeds to determine seed number per fruit. Average seed weight per seed was measured by weighing seeds on a Mettler UMT2 Ultra Microbalance. Total fitness was estimated as the average seed number per fruit multiplied by total fruit number produced per plant. All raw data is available in Appendix 1.

### Statistical analysis

All analyses were carried out in SPSS version 23 (IBM Corp.). A one-way ANOVA was used to test whether accessions differ significantly in days to flower, leaf number at flowering and growth rate. Growth rate was measured as the number of rosette leaves at bolting divided by the number of days between germination and bolting. As growth rate varies throughout rosette development, we also compared growth rate 19 days after planting. Since all of these variables were measured prior to the removal treatment, and a two-way ANOVA confirmed that none of them were affected by treatment (see below), all 10 replicates/accession were included in this set of analysis.

A two-way ANOVA was employed to analyse the effects of accession (random variable) and treatment (fixed variable) on inflorescence traits (height and number of branches), fitness components (average seed weight, average seed number per fruit, total number of fruits produced per plant, and total fitness), and senescence.

We used the ratio of the average total fitness for plants in the removal group relative to the control group for each accession, to estimate the contribution of inflorescence photosynthetic activity to fitness (from here on called “fitness maintenance”). For example, accessions that can maintain 100% of the fitness observed in control plants, despite having their leaves removed, would obtain a fitness maintenance value of 1. To investigate which factor(s) better explain variation in fitness maintained among accessions, we carried out a number of multiple regression models with fitness maintenance as the dependent variable. The independent variables included the average spring temperature in the location of origin of each accession, total leaf area, flowering time, inflorescence height, and branch number.

A caveat of our experimental design is that if rosette leaves significantly contribute to the fitness output of a plant, removal of larger rosettes might increase the cost of the leaf removal treatment. This could lead to some of the variation in fitness maintenance being due to variation in the cost of the leaf removal treatment. To address this caveat, we estimated the Pearson’s bivariate correlation between all variables measured among individual plants in the control and removal treatments, as well as correlations among accession average trait values. If the removal of leaf size affected fitness maintenance we expect that rosette size would be positively correlated with fitness under control condition, but negatively correlated in the removal treatment.

## Results

### Natural variation in vegetative growth and time to reproduction

**Fig 1:**
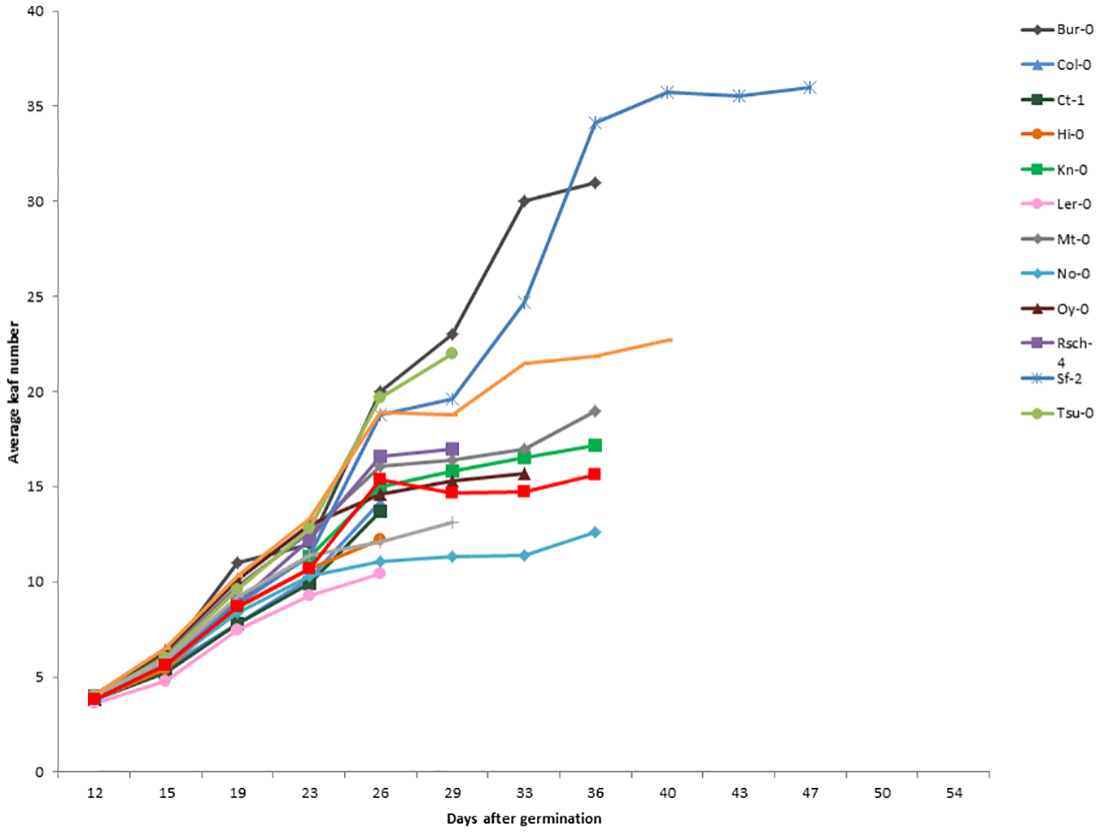
Natural variation among 15 accessions (10 replicates/accession) in vegetative growth curve as measured in terms of leaf number over time.

There is extensive variation among the accessions studied in terms of their rate of vegetative growth, rosette size and time to reproduction (Fig 1, Table 1). Significant differences in growth rate across the 15 accessions were observed when measured across the entire vegetative stage (F_14,142_ = 14.7, p<0.001), or during the first 19 days of development (F_14,142_ = 9.1, p<0.001). Accessions also significantly differ in the number of days they take to transition into the reproductive phase (F_14,142_ = 11.6, p<0.001) and in final vegetative size (measured in terms of total leaf number at transition to reproduction, F_14,142_ = 17.8, p<0.001).

Early flowering does constrain vegetative growth, since days to flowering is strongly and positively correlated with final vegetative size measured as total rosette leaf number (Table 2). We found no evidence of faster growth rate to compensate for earlier flowering. On the contrary, the tendency is for growth rate to be positively associated with flowering time (Table 2). In other words, accessions that flower earlier also tend to have slower growth rate; e.g. Ler-0 has the lowest growth rate and earliest flowering time, as shown in Table 1.

**Table 2:** Bivariate Pearson’s correlation among all variables measured. Values above the diagonal are for plants in the control group and values bellow the diagonal are for plants in the removal group. The statistical significance of the correlation is indicated by asterisks: * indicates p<0.05; and ** indicates p<0.01.

	Days to flower	Growth@ 18–20 days	Growth @ flowering	Total nr. leaves	Total leaf area	Branches	Inflorescence height	Senescence	Number of fruits	Average seed weight	Nr. Seeds/pod	Total seed nr.
Days to flower		.150	.514**	.679**	NA	0.069	.239*	.489**	-.231*	.282*	-.299*	-.326**
Growth@ 18–20 days	.045		.567**	.400**	NA	0.169	.381**	.147	.076	.135	-0.173	-0.020
Growth @ flowering	.572**	.582**		.951**	NA	.274*	.400**	.327**	-0.02	.337**	-.238*	-0.112
Total nr. leaves	.733**	.367**	.941**		NA	0.224	.362**	.491**	-0.075	.417**	-.334**	-0.214
Total leaf area	.690**	.431**	.793**	.837**		NA	NA	NA	NA	NA	NA	NA
Branches	0.057	.449**	.439**	.252*	.484**		.301**	-0.152	.466**	-0.048	.254*	.487**
Inflorescence height	0.079	.260*	.246*	0.145	.352**	.374**		0.152	.447**	0.028	0.005	.324**
Senescence	.661**	-0.063	.464**	.657**	.527**	-0.077	-0.052		0.095	.289*	-.407**	-0.184
Number of fruits	-.400**	0.108	-0.156	-.274*	-0.129	.358**	.484**	-.285*		-0.126	0.084	.791**
Average seed weight	.249*	0.171	.282*	.327**	.438**	0.172	0.08	.455**	-0.054		-.314**	-.255*
Nr. Seeds/pod	-.244*	-0.001	-.242*	-.326**	-0.191	0.199	0.218	-.482**	.302*	-.376**		.647**
Total seed nr.	-.345**	0.122	-0.19	-.314**	-0.140	.383**	.460**	-.417**	.866**	-0.21	.713**	

### Effect of rosette removal

On average, removal of rosette leaves at flowering time had a negative impact on reproductive output (Table 3). Across all accessions, rosette removal significantly reduced inflorescence height (reduced on average by 12%), number of fruits per plant (reduced on average by 38%), and total fitness (reduced on average by 40%), as shown by statistically significant effects of treatment on a two-way ANOVA (Table 3).

**Table 3:** Comparison of estimated mean and standard error for each measured trait in control and treated (leaves removed at flowering) plants. Last three columns show the F statistic and their associated probability (in brackets) from the two-way ANOVA to assess the effect of rosette removal (Treatment) and Accession on measured traits. Treatment is a fixed variable and accession is a random variable. Values in Bold indicate statistically significant effects (i.e. probability <0.05).

	Control		Removal		Treatment	Accession	Treatment*Accession
	Mean	SE Mean	Mean	SE Mean			
Flowering	32.4	0.35	32.9	0.49	1.4 (0.232)	**11.6** (<0.001)	0.5 (0.955)
Growth	0.69	0.02	0.68	0.02	1.0 (0.327)	**15.1** (<0.001)	0.9 (0.553)
Height	42.7	0.58	37.0	0.61	**39.1**(<0.001)	**13.9** (<0.001)	**2.1** (0.019)
Nodes	4.3	0.14	4.2	0.15	0.2 (0.665)	**12.1** (<0.001)	0.5 (0.925)
Branches	5.3	0.14	4.9	0.15	3.0 (0.087)	**5.3** (<0.001)	**2.6** (0.003)
Senescence	52.3	0.32	49.2	0.34	1.5 (0.225)	**5.1** (<0.001)	1.0 (0.514)
Fruit number	164.2	4.42	102.4	4.66	**93.7** (<0.001)	**4.3** (<0.001)	**2.7** (0.002)
Seeds/pod	52.1	1.18	49.2	1.24	2.9 (0.092)	**4.9** (<0.001)	0.9 (0.529)
Seed weight	22.4	0.46	21.2	0.48	3.1 (0.079)	**8.7** (<0.001)	0.8 (0.637)
Total fitness	8587.6	268.92	5116.5	283.32	**79.0** (<0.001)	**6.4** (<0.001)	**2.3** (0.009)

**Fig 2:**
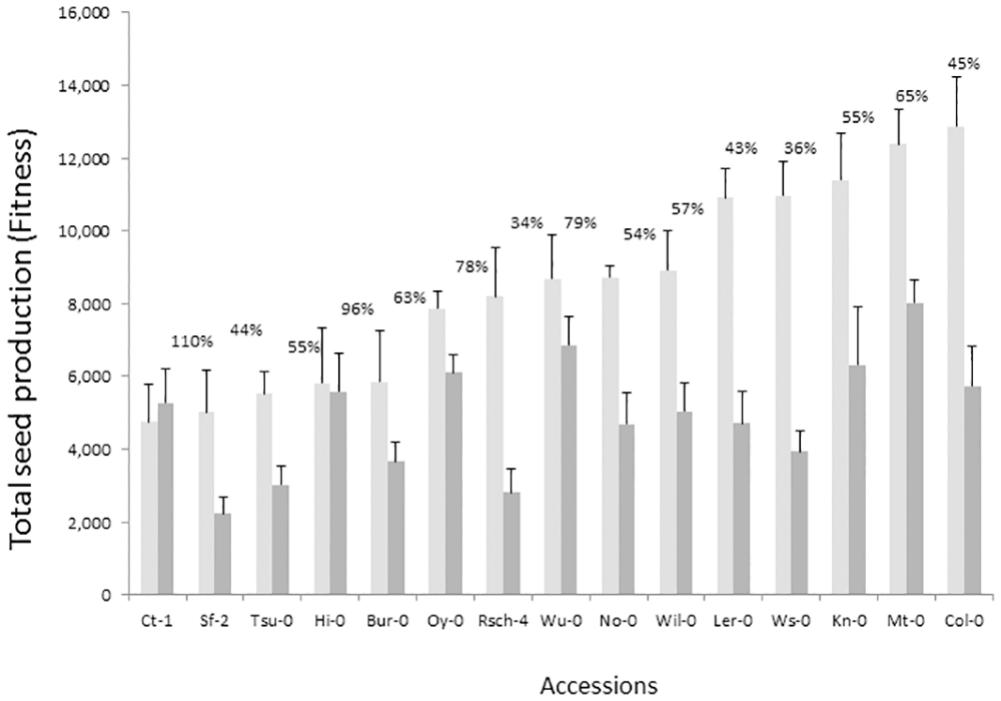
Average fitness for control (light grey) and treated (dark grey) plants for each accession. Accessions are listed in the X axis in order of their total seed production under control conditions. Numbers on the top of the bars indicate percentage of fitness maintained by inflorescence photosynthesis only (fitness maintenance).

Although there is only a marginally significant effect of rosette removal on branch number, a significant interaction between treatment and accession was observed, indicating that the effect of rosette removal on reducing branch number depended on the specific accession. An interaction between accession and treatment was also observed for inflorescence height, fruit number and total fitness. Such interaction can be clearly seen in Fig 2, where the majority of accessions displayed a large reduction in fitness with rosettes removed, but Ct-1 and Hi-0 treated plants produced almost as many or more seeds than control plants. Given that treatment plants relied solely on inflorescence photosynthesis, the ability of the inflorescence to maintain normal levels of fitness varied among accessions.

### Possible explanations for variation in inflorescence contribution across accessions

Because the two-way ANOVA (Table 3) indicated that treatment affected the number of branches and the inflorescence height, the best estimate of the accession characteristic trait value is the average number of branches and height in the control treatment. In addition, the fact that inflorescence traits respond to the leaf removal treatment differently depending on the accession, open the possibility that fitness maintenance in our experiment is not just a function of inflorescence size, but also of the specific plastic response expressed by the different accessions in response to the treatment. Thus, we also included in our regression models the ratio of the means of inflorescence traits in the two treatments to estimate this plasticity.

Models that included both number of branches and inflorescence height had colinearity problems, so we used only branches as an estimate of inflorescence size in the following model: Fitness maintenance = Average spring temperature + Flowering time + Branch number in Control treatment + Ratio of branches (number of branches in removal treatment/ number of branches in the control treatment). The full version of this model (Table 4) shows a significant effect of flowering time and branch ratio on fitness maintenance. Running forward or backward selection does not affect the model outcome. The standardized regression coefficients indicate that branch ratio has the biggest impact, with accessions that maintain or increase the number of branches after the removal of leaves having higher fitness maintenance. Flowering time has a negative coefficient, indicating that early flowering plants maintain higher fitness after leaf removal. No evidence was found that variation in fitness maintenance can be explained by the mean spring temperature of the accession’s place of origin (Table 4 and S1 Table). A model using height as an indicator of inflorescence size show similar results but those models have a lower adjusted R than the ones using branches (see S1 and S3 Tables, Supporting Information).

**Table 4:** Multiple linear regression model for fitness maintenance after leaf removal. “Spring Temperature” is the average spring temperature in the place of origin of each accession, “Control branches” is the average number of branches produced per accession in the control treatment (an indicator of inflorescence size), and branch ratio is the average number of branches in the removal treatment divided by the number in the control treatment. “Std β” stands for standardized regression coefficient, “p” for probability.

	Enter Full Model	
	R^2^ = 0.82; p<0.001	
	Std β	p
Spring Temperature	0.10	0.51
Flowering Time	-0.34	0.03
Control Branches	0.26	0.21
Branch Ratio	1.01	>0.01

A possible caveat of our experimental design is that leaf removal might introduce a negative impact of rosette size. This is because if plants with bigger rosettes have more resources for seed production, it follows that the removal of bigger rosettes might cause larger reduction in fitness. Although a significant negative correlation between fitness and leaf area is observed among plants in the removal treatment (see Table 2), there is also evidence for a negative correlation between rosette size and fitness on the control treatment. Leaf area was only measured on the removal treatment because it requires destructive sampling. However, leaf area is strongly and positively correlated with leaf number (0.941, Table 2), and leaf number is negatively correlated with fitness as strongly in the control as in the removal treatment. In addition, accession level correlations are negative and non-significant between leaf area and both control and removal treatment fitness (Pearson’s correlation = -0.26 and -0.33, respectively). Thus, it is unlikely that the negative impact of rosette size on fitness is only due to the removal treatment.

## Discussion

Here we show that flowering time does constrain vegetative growth, but that such constraint does not affect reproductive output in a predictable way. The observed decoupling between vegetative size and reproductive output is likely due to a significant contribution of inflorescence photosynthesis, which was able to maintain a significant proportion of fitness in the plants with leaves removed. It is unlikely that carbon storage could explain the results because carbon storage in *A*. *thaliana* occurs mostly in leaves, and is mainly transitory, to cope with night time lack of irradiance for photosynthesis [33]. Thus, the significant contribution of the inflorescence photosynthetic activity to the plant’s reproductive output can potentially decouple the commonly assumed trade-off between age of reproduction and fitness [3,4,5,6].

Variation in growth rate has also been suggested as a possible mechanism to decouple size, age at reproduction and reproductive output [2,18,20]. Although we did find variation in growth rate among the accessions studied (from 0.5 to 0.7 leaves per day, Table 1), we find that flowering time still correlates strongly with final rosette size. This result suggests that variation in growth rate was not sufficient to break down the constraint exerted by flowering time on vegetative size. The maintenance of the correlation between flowering time and rosette size, despite variation in growth rate occurs because the variation in growth rate is not associated in a way that allows compensation for earlier flowering; on the contrary, our results reveal earlier flowering plants tend to have a slower growth rate (Fig 1, Table 1). Our results are in agreement with Weis *et al*. [34], who found that growth rate variation had little effect on the shape of selection on flowering time.

We find that inflorescence photosynthesis contribute considerably to fitness (on average 60%), with some accessions showing no reduction in fitness despite the removal of leaves. It is noteworthy that fitness reduction was not accompanied by a significant reduction in the average seed weight (Table 3); confirming the hypothesis that seed size is highly canalized [35] despite being heritable among genotypes (Gnan et al, 2014). While previous studies have shown that reproductive structures in plants are photosynthetically active [23,26,27,36], a cost of early reproduction is still implicit in most models of life-history evolution [25,34,37,38,39]; and trade-offs between size and age of reproduction are commonly used as an explanation for lack of response to selection for larger vegetative size [40] or the maintenance of variation in flowering time [8]. Our results show that despite the constraint exercised by earlier flowering on plant vegetative size, a cost of early reproduction should not always be assumed, since reproductive structures can sustain significant proportions of the reproductive output. Field experiments imposing artificial herbivory in *A*. *thaliana* found that herbivory after flowering affects seed production less than before flowering [41], which might be due to the inflorescence significantly contributing to reproductive output, as found in this study. In addition, a field experiment with *Brassica rapa* has shown that the commonly observed selection for early flowering, is mediated by the Julian calendar not the developmental age of the plant [38]. This suggests that independent of size, plants that flower at a certain time of the year have higher fitness. Thus, smaller vegetative size does not seem to constraint fitness of *B*. *rapa* under field conditions either.

The ability to maintain fitness while relying solely on the inflorescence photosynthetic activity varies among accessions. After leaf removal, accessions maintained between 34% and 110% of the fitness of control plants (Fig 2). Earley *et al*. [27] suggested that variation among accessions in inflorescence photosynthetic activity might be adaptive. They hypothesized that shifting carbon acquisition to inflorescences may be beneficial for accessions in warmer climates, because with increasing temperatures, photosynthesis would be more efficient away from the ground surface. We addressed this possibility by testing whether the average spring temperatures from the accession’s place of origin or flowering time, was associated with fitness maintenance. While we found no evidence that spring average temperatures in their place of origin explain the variation in fitness maintenance (Table 4), we recognize that there are many other climate variables that might also be relevant and were not tested (e.g. precipitation). Flowering time in *A*. *thaliana* has been previously shown to be correlated with climate gradients and can serve as a good indicator of general ecological conditions experienced during past evolution [30,42,43]. Furthermore, Earley et al [27] hypothesis specifically suggest that environmental conditions unfavourable for photosynthesis at the soil surface (where rosette leaves are located) should select for earlier transition into reproduction to provide photosynthesis under cooler air temperatures. Thus, the fact that flowering time explains some of the variation in fitness maintenance is compatible with the idea that earlier reproduction can be an adaptive strategy to better cope with stressful climatic conditions by shifting much of the carbon fixation towards the inflorescence. The fact that rosette leaf area is strongly and negatively correlated with flowering time (Table 2), and that models that include leaf area instead of flowering time find that accessions with smaller rosettes maintain more of their fitness after leaf removal (S2 Table), indicates that it is also possible that early flowering accessions have inflorescences that contribute more to fitness because they have been selected to overcompensate for small rosettes.

Variance in fitness maintenance was also shown to be explained by inflorescence size plasticity (Table 4). Some accessions (e.g Ct-1) produced inflorescences with the same height and more branches after leaf removal than in the control treatment; while most (e.g. Ler) show reduced height and number of branches after leaf removal. The reduction in inflorescence size can be explained by the fact that resources were significantly reduced by the leaf removal treatment during inflorescence development. The accessions that maintained or improved their inflorescence size were the ones that also maintained most of their fitness. The mechanism that allows some accession to grow the same size inflorescences despite the lack of leaves is unknown, but the inflorescence of accession Mt-0 has been shown to contribute 93% of the carbon gain during the plant lifetime [27]. In agreement, Mt-0 is one of the accessions that maintained the same size infloresecence in the removal or control treatments. Thus, it is possible that accession with inflorescence with more efficient photosynthetic rates will be able to maintain their inflorescence size better. It is also possible that our manipulative approach may have triggered some stress responses akin to responses to herbivory, and that accessions from locations with higher herbivory recovered better. Genetic variation in tolerance to herbivory have been previously observed [44,45], and a relationship between inflorescence architecture and tolerance to herbivory has been documented across a number of species [46]. Unfortunately, to the best of our knowledge, a mechanism through which such stress could be measured has not been characterized, and a previous study that attempted to identify quantitative trait locus underlying tolerance to herbivory was unsuccessful [44].

In summary, our manipulative approach shows unequivocally that the photosynthetic activity of the inflorescence of *A*. *thaliana* is capable of maintaining a high proportion of the plant’s fitness. While similar experiments need to be carried out in more species to confirm the generality of our results, evidence in *Lolium perenne* [40] and *B*. *rapa* [34] support the conclusion that assuming an inherent cost of early reproduction due to exclusive contribution of vegetative tissues to resource acquisition might not be warranted. On the contrary, our results suggest that earlier transition into reproduction can diversify life-history strategies, and might be favoured depending on environmental conditions, such as locations where soil temperatures are less optimal for photosynthesis, or where herbivory or competition compromise the ability to maintain high carbon fixation through rosettes.

## Supporting information

S1 File Raw data. (CSV)

S2 File Average trait value per accession. (XLSX)

S1 Table Modelling of fitness maintenance, including the average inflorescence height and height plasticity (average height in removal treatment/ average height in the control treatment). “Std β” stands for standardized regression coefficient, and “p” is the probability associated with each factor. Bold p values indicate significant results.
(DOCX)

S2 Table Modelling of fitness maintenance, including leaf area instead of flowering time. “Std β” stands for standardized regression coefficient, and “p” is the probability associated with each factor. Bold p values indicate significant results.
(DOCX)

S3 Table Modelling of fitness maintenance, including leaf area instead of flowering time. “Std β” stands for standardized regression coefficient, and “p” is the probability associated with each factor. Bold p values indicate significant results.
(DOCX)
