# Supplementary material for: Inflorescence photosynthetic contribution to fitness releases Arabidopsis thaliana plants from trade-off constraints on early flowering
Source: PLoS One. 2017 Oct 3;12(10):e0185835. doi: 10.1371/journal.pone.0185835 (PMC5626516; doi:10.1371/journal.pone.0185835)
Supplement: S1 Table — “Std β” stands for standardized regression coefficient, and “p” is the probability associated with each factor. Bold p values indicate significant results. (DOCX) [file pone.0185835.s003.docx]

**S1 Table –** Modelling of fitness maintenance, including the average inflorescence height and height plasticity (average height in removal treatment/ average height in the control treatment). “Std β” stands for standardized regression coefficient, and “p” is the probability associated with each factor. Bold p values indicate significant results.

|  | Enter Full Model | | |
| --- | --- | --- | --- |
|  | R^2^=0.57; p=0.01 | | |
|  | Std β |  | p |
| Spring Temperature | 0.11 |  | 0.58 |
| Flowering Time | -0.06 |  | 0.81 |
| Control Height | 0.07 |  | 0.78 |
| Height Ratio | 0.82 |  | **0.002** |
