# Supplementary material for: Inflorescence photosynthetic contribution to fitness releases Arabidopsis thaliana plants from trade-off constraints on early flowering
Source: PLoS One. 2017 Oct 3;12(10):e0185835. doi: 10.1371/journal.pone.0185835 (PMC5626516; doi:10.1371/journal.pone.0185835)
Supplement: S2 Table — “Std β” stands for standardized regression coefficient, and “p” is the probability associated with each factor. Bold p values indicate significant results. (DOCX) [file pone.0185835.s004.docx]

**S2 Table –** Modelling of fitness maintenance, including leaf area instead of flowering time. “Std β” stands for standardized regression coefficient, and “p” is the probability associated with each factor. Bold p values indicate significant results.

|  | Enter Full Model | | |
| --- | --- | --- | --- |
|  | R^2^=0.85; p<0.001 | | |
|  | Std β |  | p |
| Spring Temperature | 0.06 |  | 0.63 |
| Leaf Area | -0.38 |  | **0.01** |
| Control Branches | 0.28 |  | 0.11 |
| Branch Ratio | 1.09 |  | **<0.001** |
