# Supplementary material for: Inflorescence photosynthetic contribution to fitness releases Arabidopsis thaliana plants from trade-off constraints on early flowering
Source: PLoS One. 2017 Oct 3;12(10):e0185835. doi: 10.1371/journal.pone.0185835 (PMC5626516; doi:10.1371/journal.pone.0185835)
Supplement: S3 Table — “Std β” stands for standardized regression coefficient, and “p” is the probability associated with each factor. Bold p values indicate significant results. (DOCX) [file pone.0185835.s005.docx]

**S3 Table –** Modelling of fitness maintenance, including leaf area instead of flowering time. “Std β” stands for standardized regression coefficient, and “p” is the probability associated with each factor. Bold p values indicate significant results.

|  | Enter Full Model | | |
| --- | --- | --- | --- |
|  | R^2^=0.57; p<0.001 | | |
|  | Std β |  | p |
| Spring Temperature | 0.10 |  | 0.58 |
| Leaf Area | -0.07 |  | 0.75 |
| Control Height | 0.08 |  | 0.73 |
| Height Ratio | 0.82 |  | **0.002** |
